# Hydroxylamine Analogue of Agmatine: Magic Bullet for Arginine Decarboxylase

**DOI:** 10.3390/biom10030406

**Published:** 2020-03-06

**Authors:** Mervi T. Hyvönen, Tuomo A. Keinänen, Gulgina K. Nuraeva, Dmitry V. Yanvarev, Maxim Khomutov, Elena N. Khurs, Sergey N. Kochetkov, Jouko Vepsäläinen, Alexander A. Zhgun, Alex R. Khomutov

**Affiliations:** 1 School of Pharmacy, Biocenter Kuopio, University of Eastern Finland, Kuopio Campus, P.O. Box 1627, FI-70211 Kuopio, Finland; tuomo.keinanen@uef.fi (T.A.K.); jouko.vepsalainen@uef.fi (J.V.); 2 Research Center of Biotechnology, Russian Academy of Sciences, 119071 Moscow, Russia; nuraevagulgina@mail.ru (G.K.N.); zzhgun@mail.ru (A.A.Z.); 3 Engelhardt Institute of Molecular Biology, Russian Academy of Sciences, Vavilov Street 32, 119991 Moscow, Russia; jdmitry@hotmail.com (D.V.Y.); hommaximus@mail.ru (M.K.); enkhurs@yandex.ru (E.N.K.); kochet@eimb.ru (S.N.K.)

**Keywords:** polyamines, arginine decarboxylase, ornithine decarboxylase, *O*-substituted hydroxylamine, nanomolar inhibitor, *Acremonium chrysogenum*, *Escherichia coli*

## Abstract

The biogenic polyamines, spermine, spermidine (Spd) and putrescine (Put) are present at micro-millimolar concentrations in eukaryotic and prokaryotic cells (many prokaryotes have no spermine), participating in the regulation of cellular proliferation and differentiation. In mammalian cells Put is formed exclusively from *L-*ornithine by ornithine decarboxylase (ODC) and many potent ODC inhibitors are known. In bacteria, plants, and fungi Put is synthesized also from agmatine, which is formed from *L-*arginine by arginine decarboxylase (ADC). Here we demonstrate that the isosteric hydroxylamine analogue of agmatine (AO-Agm) is a new and very potent (*IC*_50_ 3•10^−8^ M) inhibitor of *E. coli* ADC. It was almost two orders of magnitude less potent towards *E. coli* ODC. AO-Agm decreased polyamine pools and inhibited the growth of DU145 prostate cancer cells only at high concentration (1 mM). Growth inhibitory analysis of the *Acremonium chrysogenum* demonstrated that the wild type (WT) strain synthesized Put only from *L*-ornithine, while the cephalosporin C high-yielding strain, in which the polyamine pool is increased, could use both ODC and ADC to produce Put. Thus, AO-Agm is an important addition to the set of existing inhibitors of the enzymes of polyamine biosynthesis, and an important instrument for investigating polyamine biochemistry.

## 1. Introduction

The biogenic polyamines, spermine (Spm) and spermidine (Spd), are organic polycations present at micro-millimolar concentrations in eukaryotic and prokaryotic cells (in many of prokaryotes Spm is absent) where they participate in the regulation of vital cellular functions including proliferation and differentiation [1,2,3]. Disturbances of polyamine metabolism have been associated with the development of many diseases, including cancer (tumor cells have elevated polyamine levels) [1,4,5,6]. Therefore, it is important to investigate the cellular functions of the feasibly interconvertible and partly interchangeable Spm and Spd. Mutant and genetically modified microorganisms and cell lines, as well as transgenic animals have been widely used for these purposes during the last decades [7]. However, there is still a need for specific inhibitors and inducers of the key enzymes of polyamine metabolism, as well as functionally active mimetics of Spm and Spd [8,9,10,11,12].

In vertebrates, ornithine decarboxylase (ODC) is the key and the rate-limiting enzyme of polyamine biosynthesis, since putrescine (Put), formed from the decarboxylation of *L*-ornithine (*L-*Orn), then gives rise to Spd and Spm [2,4]. Many potent ODC inhibitors are currently known [8,12]. α-(Difluoromethyl)ornithine (DFMO) was synthesized in 1978 [13], and is the best-known and most widely used of the ODC inhibitors, serving for many years as the “gold standard inhibitor” of polyamine research [8,12]. DFMO is used as a drug to cure African sleeping sickness [14] and has also advanced in clinical trials with promising results as a cancer chemopreventive agent among populations with an elevated risk for specific epithelial cancers [15]. In addition, DFMO has fungicidal activity; this was demonstrated for the first time when it conferred protection on bean plants against infection by uredospores of the bean rust fungus *Uromyces phaseoli Linnaeus* in greenhouse experiments [16].

ODC is a pyridoxal-5’-phosphate (PLP) dependent enzyme, and among the carbonyl reagents typically inhibiting this class of enzymes, 1-aminooxy-3-aminopropane (APA), an isosteric hydroxylamine-containing analogue of Put, was found to inhibit mammalian ODC at nanomolar concentrations [17,18]. In some cases, APA was even more potent than DFMO [19,20,21]. Interestingly, in the case of phytopathogenic fungi *Pyricularia oryzae Cav.* only APA, but not DFMO bleached the mycelium, despite the fact that both exhibited fungicidal activity [22]. The biosynthesis of Put is more variable in bacteria, plants and fungi than in vertebrates [3]. In many cases, Put is synthesized from *L-*arginine (*L-*Arg), which is first decarboxylated to agmatine (the rate-limiting step in Put biosynthesis) by the biosynthetic arginine decarboxylase (ADC; also known as ArgDC, gene name *SpeA* for *E. coli* [3,23]). Agmatine is then hydrolyzed by agmatinase to yield Put and urea. ADC is a PLP-dependent enzyme, but it shares only weak sequence homology to other PLP-dependent decarboxylases, including ODC. The holoenzyme of *E. coli* ADC is a tetramer, having one molecule of PLP bound to each 70-kDa subunit, and the X-ray crystallographic data for *E. coli* ADC is available [24]. *E. coli* contains also an acid-inducible arginine decarboxylase (*AdiA*), which is an essential component in the arginine-dependent acid-resistance system [25]. The spectrum of the ADC inhibitors is not as extensive as in the case of ODC. The most well-known of these molecules is *D,L*-α-(difluoromethyl)arginine (DFMA) [26], which has been successfully used to inhibit ADC from different microorganisms and plants [27,28,29,30]. 

In the present paper we have expanded the family of ADC inhibitors to include 1-aminooxy-3-guanidinopropane (aminooxyagmatine, AO-Agm), which is a hydroxylamine-containing isostere of agmatine and which very efficiently and pseudo-irreversibly inhibited the biosynthetic ADC from *E. coli* having an *IC*_50_ value of 30 ± 1 nM. AO-Agm was almost two orders of magnitude less efficient towards ODC from *E. coli* i.e., confirming the specificity of its inhibitory activity. Growth inhibitory analysis (effects of AO-Agm, DFMA, APA and DFMO) provided evidences that on the contrary to the wild-type (WT) strain of *Acremonium chrysogenum*, which used *L-*Orn as a precursor of Put, in the high-cephalosporin-yielding (HY) strain both ODC and ADC may contribute to the biosynthesis Put, the precursor of the polyamines.

## 2. Materials and Methods 

### 2.1. Materials 

DFMA was purchased from Santa Cruz Biotechnology Inc. AO-Agm was synthesized as described previously [31] and APA was prepared following published protocol [32]. DFMO was a kind gift from Prof. P.M. Woster (Medical University of South Carolina, Charleston, SC, USA). *L*-Arg, *L*-Orn, Put, Spd, agmatine, 1,7-diaminoheptane, acetonitrile, tetrahydrofuran and 5-(dimethylamino)naphthalene-1-sulfonyl chloride (Dans-Cl) were obtained from Sigma-Aldrich. [^14^C]-*L*-Orn was purchased from GE Healthcare. DU145 prostate cancer cells were obtained from American Type Culture Collection. 

### 2.2. Preparation of Partially Purified ADC and ODC

The purification was performed using a modified procedure of earlier publication [33]. *E. coli* strain DH5α was grown in Luria-Bertani broth at +37 °C with shaking (200 rpm) overnight. The cells were collected by centrifugation and the pellet was homogenized with Potter-Elvehjem in 10 volumes (*w/v*) of ice-cold 50 mM Tris-HCl pH 7.4, 1 mM DTT. The cell debris was removed by centrifugation at 12,000 g for 30 min at +4 °C. Solid (NH_4_)_2_SO_4_ was added to the supernatant, and the pellet precipitating between 39% and 53% saturation was separated. The precipitate was resuspended in ice-cold buffer containing 50 mM Tris-HCl pH 7.4 and 1 mM DTT and desalted by passage through Sephadex G-25 column (NAP-10 column, GE Healthcare). The protein concentration was measured with Bio-Rad protein reagent, and the partially purified enzyme preparation was mixed with 20% glycerol and aliquoted for storage at −80 °C. 

### 2.3. E. coli ADC and ODC Activity Assays

The ADC reaction mixture contained 50 mM Tris-HCl pH 7.8, 4 mM MgCl_2_, 1 mM DTT, 1.0 or 0.1 mM PLP and 20 μg of partially purified enzyme preparation in a total volume of 162 μL. The ODC reaction mixture contained 50 mM Tris-HCl pH 7.8, 1 mM EDTA, 2.4 mM DTT, 0.1 mM PLP and 20 μg of partially purified enzyme preparation. The mixtures were either preincubated or not preincubated for 15 min at +40 °C in the presence of the inhibitor, after which the reaction was started by the addition of 18 μL of 10× substrate (L-Arg for ADC and L-Orn for ODC). After incubation for 30 min (ADC) or 20 min (ODC) at +40 °C, 20 μL of 50% sulphosalicylic acid containing 50 μM diaminoheptane (internal standard) was added and the samples were analyzed for the content of agmatine (ADC) and Put (ODC) with HPLC as described previously [34]. The kinetic parameters and *IC*_50_ values were determined with the aid of GraphPad Prism version 5.03 using nonlinear regression analysis. 

### 2.4. Ultrafiltration of ADC~PLP=AO-Agm Complex

Partially purified enzyme preparation was preincubated for 15 min with 15 μM AO-Agm (which produced 100% inhibition) or H_2_O (control) and 0.1 mM PLP in the assay buffer. The samples were placed on ice, and half of the reactions were filtered through a 3K cutoff ultrafiltration device (Millipore) and washed twice with 600 μL of buffer containing 50 mM Tris-HCl pH 7.4, 1 mM DTT and 0.1 mM PLP. ADC activity was then assayed.

### 2.5. Assay of Mouse Kidney ODC Activity

C57Bl/6J male mouse kidney was homogenized with TissueLyzer (Qiagen) to 2 volumes of buffer containing 50 mM Tris-HCl pH 7.4, 1 mM DTT, 1 mM EDTA, 0.1% Triton X-100 and 1× Complete EDTA-free protease inhibitor cocktail (Roche) and centrifuged at 12,000 g for 30 min at +4 °C. Aliquots of the supernatant were mixed with 1–5000 μM of AO-Agm and the ODC assay was carried out as previously described [35]. The reaction mixture contained 0.4 mM PLP and 0.4 mM *L*-Orn.

### 2.6. Cell Culture

DU145 prostate cancer cells were cultured in high-glucose Dulbecco’s Modified Eagle’s Medium supplemented with 10% heat-inactivated fetal bovine serum, 2 mM *L*-glutamine and 50 μg/mL gentamycin (all from Sigma-Aldrich). The cells were plated on 6-well plates and allowed to attach overnight. The cells were treated for 1 day with 10–1000 μM AO-Agm in the presence or absence of semicarbazide (SC), an inhibitor of serum amine oxidases. The cells were harvested, and the pellet was mixed with 150 μL of 5% sulphosalicylic acid containing 5 μM diaminoheptane (internal standard). Polyamine analysis was carried out as described previously [34].

### 2.7. Fungal Growth Inhibition

Two strains of *Acremonium chrysogenum* were used in this study: the wild-type (Brotzu) isolate ATCC 11550 (WT) [36] and high-cephalosporin-yielding RNCM F-4081D (HY), derived from the WT [37]. The cultivation conditions were the same as previously described [38,39]. The filamentous fungi were cultivated on agarized Czapek-Dox (CD) medium (30 g/L sucrose, 2 g/L NaNO_3_, 1 g/L K_2_HPO_4_, 0.5 g/L MgSO_4_×7 H_2_O, 0.5 g/L KCl, 0.01 g/L FeSO_4_×7 H_2_O, 20 g/L agar, pH 7.0–7.4). To determine the toxic effect of AO-Agm, APA, DFMA and DFMO on the growth of fungal cells the drop-dilution method was used with some modifications as described earlier [40,41]. Cells were collected from agar slants and diluted with 0.9% NaCl solution up to *OD*_600_ = 0.5 (basic concentration), followed by serial tenfold dilutions with the same solution. Then, 2 μL of cell suspension was inoculated onto Petri dishes with CD medium prepared with or without the addition of the inhibitors (AO-Agm, APA, DFMA and DFMO), each at 5 mM concentration. The inoculated Petri dishes were incubated for 21 days at 26 °C. The inhibitory effects of the compounds were measured every three days after inoculation and evaluated by measuring the ratio of colony growth on agarized CD medium supplemented with AO-Agm, APA, DFMA, or DFMO to the growth in the control (agarized CD medium without any additions). The formula: growth inhibition % = [(Dc − Dt)/Dc] × 100, where Dc indicates the colony diameter in control set, and Dt indicates the colony diameter in the treatment set that was used to determine the percent of fungal growth inhibition. The data recorded were measured in triplicate and repeated at least twice.

### 2.8. Determination of Polyamine Content in the Fungi

WT and HY *A. chrysogenum* strains were preliminarily grown in tubes on CD medium slants for 7 days at 25 °C and used for the inoculation of 30 mL of a liquid CD medium (seed medium). The strains were cultivated on CD medium for 48 h at 26 °C and inoculated into ten volumes of CD medium. The fermentation was carried out for 120 h at 26 °C in 250-mL Erlenmeyer flasks on a CERTOMAT BS-1 shaker (Sartorius, Germany) at 230 rpm, as described earlier [42]. After 24 h of culture, 1 mL aliquots were removed, fungi were separated by centrifugation (15 min, 14,000 g) and washed with H_2_O (3 × 2 mL). The washed biomass was subjected to three cycles of freezing (−80 °C) and thawing at 20 °C in 5% perchloric acid. After the final thaw, samples were vortexed for 2 min and centrifuged for 10 min at 14,000 g. The supernatant was used for the determination of polyamine content.

The dry biomass was prepared from 2 mL aliquots of fermentation media after 24 h. The biomass was separated by centrifugation (15 min, 14,000 g), the precipitated fungi cells were washed in triplicate with 10 volumes of H_2_O, dried at 80 °C for 96 h to a constant weight and used for normalization of polyamine content in the fungi. 

Polyamines were determined by HPLC from 50 µL of the 5% perchloric acid supernatant using a precolumn modification with Dans-Cl following mostly the published protocol [43]. 1,7-Diaminoheptane was used as an internal standard and proline applied to quench the dansylation reaction. The solution of the dansylated polyamines in toluene (2 μL) was mixed with 50% aq. ethanol (13 µL) and applied on a reversed phase column (Cosmosil C18-MS-II, 250 × 4.6 mm, 5 µm). The column was eluted (1 mL/min) with the gradient: 0 min—0% B; 4 min—65% B; 17 min—65% B; 19 min—100% B, 23 min—100% B, 25 min—0% B; 30 min—0% B. System A—40% acetonitrile, 60% H_2_O. System B—80% acetonitrile, 20% tetrahydrofuran. Column temperature 40 °C, pressure 80–120 bar, fluorescent detection: *λ_ex_* 340 nm, *λ_em_* 530 nm (detector RF-20A, Shimadzu Scientific Instrument, Columbia, MD, USA). 

## 3. Results

### 3.1. Inhibition of E. coli ADC and ODC by AO-Agm

The kinetic parameters determined for the partially purified biosynthetic ADC from *E. coli* were *V_max_* = 546 ± 5 nmol/h/mg protein and *K_m_* = 158 ± 5 μM for *L-*Arg (Supplementary Figure S1a). For ODC, they were *V_max_* = 709 ± 7 nmol/h/mg protein and *K_m_* = 7.2 ± 0.3 mM for *L-*Orn (Supplementary Figure S1b). 

**Figure 1:**
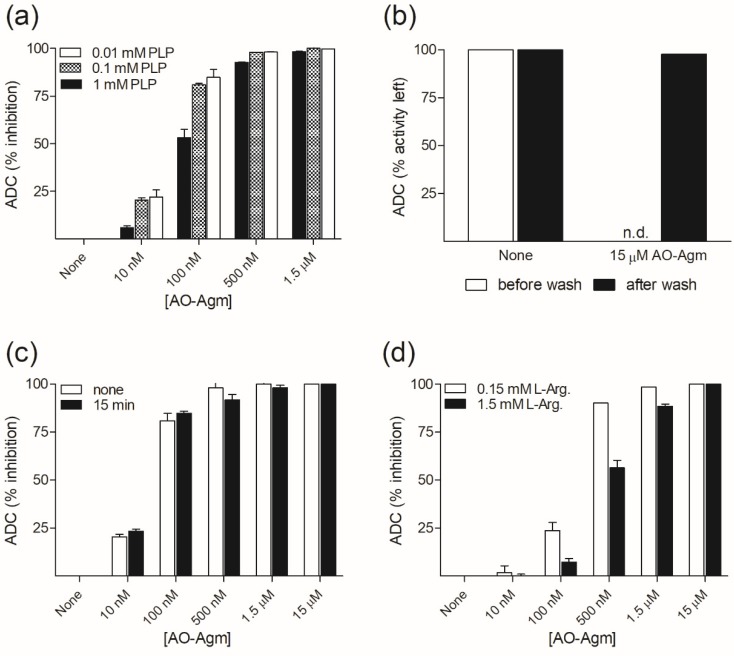
Inhibition (%) of *E. coli* ADC by AO-Agm. (**a**) Effect of different PLP concentrations (reaction conditions: *L*-Arg 150 μM = *K_m_* and 15 min preincubation of the enzyme with AO-Agm in the absence of *L*-Arg). (**b**) Effect of ultrafiltration on the stability of ADC~PLP=AO-Agm inhibitory complex (reaction conditions: 0.1 mM PLP, 15 μM AO-Agm, incubation for 15 min and then ultrafiltration and washings with a buffer containing 0.1 mM PLP). (**c**) Effect of preincubation time (reaction conditions: 0.1 mM PLP and 150 μM *L*-Arg). (**d**) Effect of different *L*-Arg concentrations (reaction conditions: 0.1 mM PLP, *L*-Arg 150 μM = *K_m_*, or 1.5 mM = 10 × *K_m_*; no preincubation, the reaction was started with ADC addition). Data are means ± SD, *n* = 3.

First, we investigated the inhibition of ADC by AO-Agm. The partially purified enzyme preparation was preincubated for 15 min with the inhibitor and varying the concentrations of PLP in the assay mixture. The reaction was started with the addition of the substrate (0.15 mM *L-*Arg, corresponding to the measured *K_m_* value at pH 7.8, while according to published data [44], the *K_m_* value for *L-*Arg is 0.03 mM at pH 8.4). With 1 mM PLP in the substrate mixture, AO-Agm had an *IC*_50_ value of 89 ± 1 nM, while lowering the PLP concentration to 0.1 mM decreased the *IC*_50_ value to 30 ± 1 nM (Figure 1a), i.e., AO-Agm was a very effective inhibitor of ADC. Lowering of the PLP concentration down to 0.01 mM did not markedly affect the determined *IC*_50_ value (26 ± 1 nM). The efficiency of the inhibition of ADC was increased when the PLP concentration in the substrate mixture was lowered. This is in agreement with the reversible character of the PLP binding to the apo-enzyme—the *K_m_* value of PLP is 0.6 μM [44]. Correspondingly, an excess of PLP can shift the equilibria (1) and (2):

*apo*-ADC + PLP ↔ *holo*-ADC + AO-Agm ↔ **ADC~PLP=AO-Agm** + PLP (1)

*ADC-Inhibitor complex*

**ADC~PLP=AO-Agm** + PLP ↔ *holo*-ADC + PLP=AO-Agm (2)

*ADC-Inhibitor complex*

to the direction of the formation of holo-ADC and liberation of PLP=AO-Agm oxime. 

Mouse kidney ODC also has a reversibly bound PLP; its *K_m_* value is 0.3 μM [45]. Gel filtration of the ODC~PLP=APA inhibitory complex, using a PLP-containing buffer for elution, restored the ODC activity [17]. This is in good agreement with the less efficient inhibition of ADC with AO-Agm when high PLP concentrations were present in the substrate mixture (Figure 1a). We found that the ultrafiltration and washing of ADC~PLP=AO-Agm inhibitory complex with buffer containing 0.1 mM PLP fully restored ADC activity (Figure 1b) indicating that AO-Agm is a very potent, pseudo-irreversible inhibitor of ADC.

Omitting the preincubation time did not significantly alter the efficiency of the inhibition (Figure 1c). In this experiment, the PLP concentration (0.1 mM) was fixed, the preincubation time was varied and the reaction was started with the addition of *L-*Arg (final concentration 0.15 mM). PLP forms a Shiff base with ε-amino group of Lys (internal aldimine) in the ADC active center and it is known that Shiff bases react much faster with *O*-substituted hydroxylamines, if compared with the reaction for the free carbonyl group [46]. These data confirmed that AO-Agm reacted directly with the internal aldimine yielding E-I complex, but not with an excess of PLP in the solution giving rise to PLP=AO-Agm oxime.

Next, we checked the ability of *L-*Arg to protect ADC from AO-Agm inhibition. In this experiment, the PLP concentration (0.1 mM) was fixed. The substrate mixtures contained either 0.15 mM, or 1.5 mM of *L-*Arg and the reaction was started with the addition of ADC, omitting the preincubation of the enzyme with the inhibitor. It can be clearly seen that when 0.15 mM *L-*Arg was initially present in the substrate mixture, AO-Agm at 100 nM inhibited the enzyme by 25%, while at AO-Agm 500 nM, the ADC was inhibited by 95% (Figure 1d). However, when 1.5 mM *L-*Arg was initially present in the substrate mixture, AO-Agm at 100 nM inhibited the enzyme only by about 5% and at 500 nM by 60% (Figure 1d). Thus, the increasing concentrations of *L-*Arg caused a competition for binding with AO-Agm and protected ADC against inhibition. These findings indicate that in the reversible step of the inhibition, AO-Agm was properly bound into the active center of the enzyme and that the guanidino group anchored the inhibitor. 

**Figure 2:**
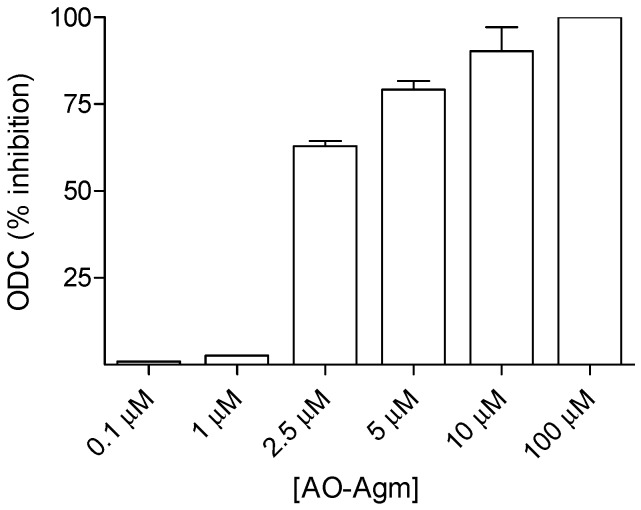
Inhibition (%) of *E. coli* ODC by AO-Agm (*L-*Orn 7 mM = *K_m_*, 15 min preincubation of the enzyme with AO-Agm in the absence of *L-*Orn, substrate mixture contained 0.1 mM PLP). Data are means ± SD, *n* = 3.

Next, we investigated the inhibition of *E. coli* ODC with AO-Agm under the same conditions as used for ADC, i.e., using 0.1 mM concentration of PLP in the substrate mixture and 15 min preincubation of the enzyme with the inhibitor. Under these conditions AO-Agm had an *IC*_50_ value of only 2.3 ± 1.1 μM towards ODC from *E. coli* (Figure 2). These data clearly demonstrated that AO-Agm is a specific and very effective inhibitor of ADC.

### 3.2. Inhibition of E. coli ADC and ODC by DFMA, DFMO and APA

**Figure 3:**
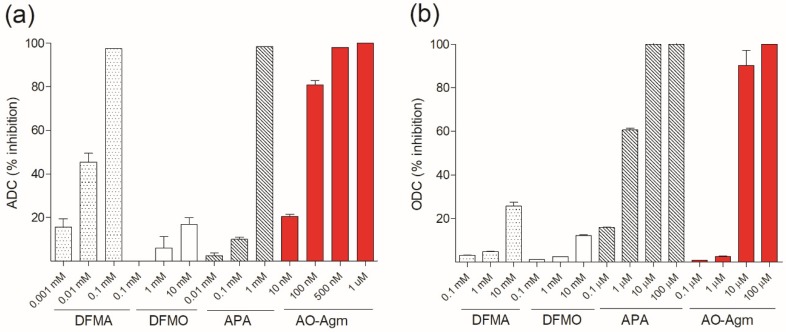
(**a**) Inhibition (%) of *E. coli* ADC by DFMA, DFMO, APA and AO-Agm (reaction conditions: 15 min preincubation of the enzyme with inhibitor in the absence of *L-*Arg; 0.1 mM PLP; reaction was started with the addition of *L-*Arg 150 μM (= *K_m_*)). (**b**) Inhibition (%) of *E. coli* ODC by DFMA, DFMO, APA and AO-Agm (reaction conditions: 15 min preincubation of the enzyme with inhibitor in the absence of *L-*Orn; 0.1 mM PLP; reaction was started with the addition of *L-*Orn 7 mM (= *K_m_*)). Data are means ± SD, *n* = 3.

The comparison of the inhibitory activities of AO-Agm towards *E. coli* ADC with that of APA, DFMA and DFMO demonstrated that AO-Agm was the most potent inhibitor (Figure 3a). In these experiments, the substrate mixture had a fixed PLP concentration (0.1 mM) and 15 min preincubation was used (in the case of DFMO and DFMA, this time is sufficient to cause irreversible inhibition). After the preincubation, the reaction was started with the addition of *L-*Arg at a final concentration of 0.15 mM. Under these conditions, the obtained *IC*_50_ value for DFMA was 10 ± 1 μM whereas AO-Agm was found to be a 330 times more potent inhibitor of ADC than DFMA. APA (nanomolar inhibitor of mammalian ODC) has the amino-, but not the guanidino group causing unfitted anchoring of APA in the active center of ADC. In our hands, APA had an *IC*_50_ value of 33 ± 2 μM towards ADC from *E. coli*, i.e., the inhibition was 1000 times weaker as compared with the effectiveness of AO-Agm towards ADC.

The comparison of the inhibitory potency of AO-Agm, APA, DFMO and DFMA towards ODC from *E. coli* showed that APA, being an isostere of Put and a carbonyl reagent, had an *IC*_50_ value of 609 ± 30 nM (0.1 mM PLP and 7 mM (=*K_m_*) of *L-*Orn, 15 min preincubation) (Figure 3b). Under the same reaction conditions, AO-Agm revealed an *IC*_50_ value of 2.3 ± 1.1 μM (Figure 2 and Figure 3b). The observed very low potency of DFMO to inhibit *E. coli* ODC is in accordance with the literature data [47] and reflects the details of the fine structure of the enzyme active center, like it has been demonstrated for *Entamoeba histolytica* ODC [48], which is also resistant towards DFMO.

### 3.3. Inhibition of Mammalian and Fungal Cell Growth by AO-Agm

We also tested the inhibitory effect of AO-Agm on mouse kidney ODC and found the *IC*_50_ value to be 5.3 ± 0.7 μM, when standard assay conditions (0.4 mM *L-*Orn (= 4 × *K_m_* for mouse ODC) and 0.4 mM PLP) were used (Supplementary Figure S2). No detectable ADC activity was found from mouse kidney lysate. 

High (1 mM) concentrations of AO-Agm moderately inhibited the proliferation of DU145 prostate cancer cells and decreased the intracellular polyamine pools, whereas lower concentrations i.e., 0.01 mM and 0.1 mM were without effect (Table 1). This was most likely related to nonspecific inhibition of ODC at high concentrations, because mammals do not have a functional ADC [49]. The addition of semicarbazide (SC), an inhibitor of serum amine oxidases that are present in the cell culture medium, potentiated the effects of AO-Agm, indicating that AO-Agm is a substrate for serum amine oxidase(s). This has been previously observed for APA, which was degraded in the cell culture medium to a level about 10% of its initial concentration in 48 h in the absence of amine oxidase inhibitor [50].

**Figure 4:**
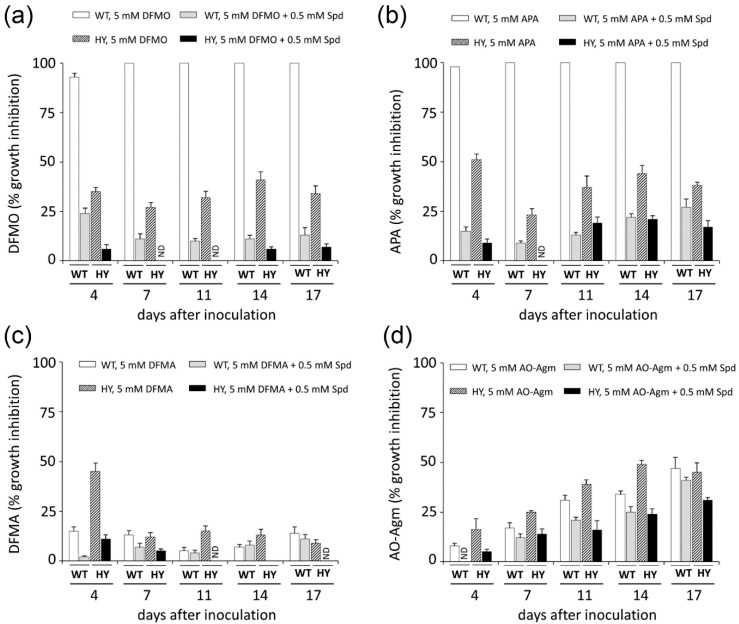
Growth inhibition (%) of *A. chrysogenum* WT and HY strains with 5 mM DFMO (**a**), 5 mM APA (**b**), 5 mM DFMA (**c**), 5 mM AO-Agm, (**d**) and reverse of the inhibition with 0.5 mM Spd (**a**–**d**). Data are means ± SD, *n* = 3. ND, not detected.

**Figure 5:**
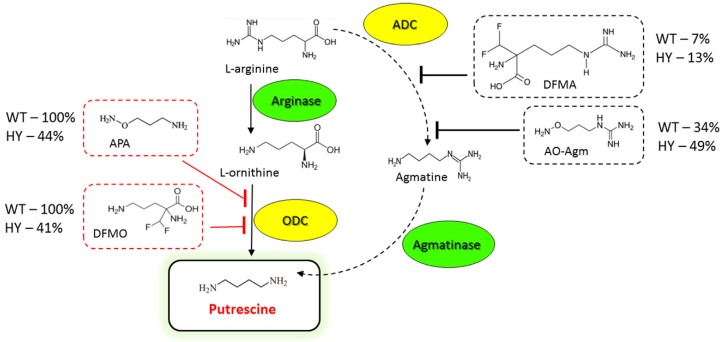
Possible pathways of Put biosynthesis in *A. chrysogenum* and the growth inhibition (%) of the WT (wild type) and HY (cephalosporin C high-yielding) strains with the inhibitors of ODC and ADC at 14 days after inoculation on CD medium.

Next, by using a growth inhibitory analysis we attempted to elucidate the pathway of Put synthesis in wild-type (WT) and cephalosporin C high-yielding (HY) strains of the filamentous fungi *A. chrysogenum*. For this purpose, the antifungal activities of the inhibitors of ADC (DFMA and AO-Agm) and ODC (DFMO and APA) were evaluated. The inhibition was assessed quantitatively by comparing the colony growth on CD agar medium. First, the effects of ODC inhibitors were examined. The growth of WT strain was completely inhibited with either 5 mM DFMO or 5 mM APA (Figure 4 and Figure 5). When 1 mM APA was used, practically 95% inhibition of the WT strain growth was observed on the 17th day after inoculation (Figure S3). By contrast, 1 mM DFMO was significantly less effective against the WT strain (Figure S3). These data demonstrated that the WT strain uses *L-*Orn as a precursor of Put and in this pathway, ODC is the rate-limiting enzyme.

The activities of DFMA and AO-Agm (ADC inhibitors) on the growth of the WT *A. chrysogenum* were not as pronounced as those of DFMO and APA. A concentration of 5 mM DFMA inhibited the growth of the WT strain by 7% on the 14th day after inoculation (Figure 4 and Figure 5); this might be due to the transformation of DFMA into DFMO inside the fungal cells, as this phenomenon has been demonstrated in both tobacco and mammalian cells [51]. AO-Agm was much more effective towards the WT strain as compared with DFMA and inhibited growth by 34% on the 14th day after inoculation (Figure 4 and Figure 5). The observed effect could be related to the nonspecific inhibition of ODC when a high (5 mM) concentration of AO-Agm was used. Similar growth inhibition of DU145 cells was also observed with a high AO-Agm concentration, and its effect on the polyamine pool resembled the effects of other ODC inhibitors such as DFMO (Table 1). Hence, the activity of the inhibitors of ADC, i.e., DFMA and AO-Agm, provided evidence that the WT *A. chrysogenum* uses *L-*Orn as a precursor of Put.

**Figure 6:**
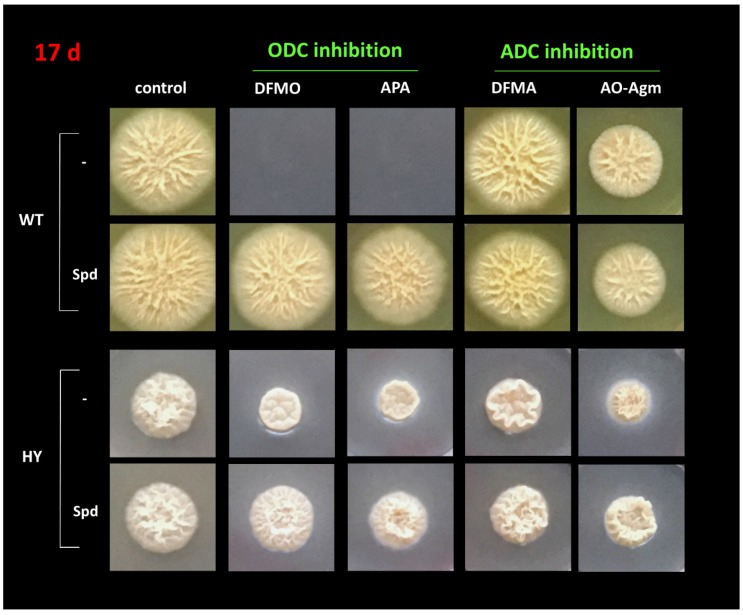
Growth inhibition of the WT and HY *A. chrysogenum* strains with 5 mM DFMO, APA, DFMA, and AO-Agm and the reversal of the inhibition with 0.5 mM Spd at 17 days after inoculation on CD medium.

The observed inhibition of the growth of the WT strain was specific and directly associated with the inhibition of polyamine biosynthesis, because addition of 0.5 mM Spd reversed the growth arrest caused by 5 mM DFMO or 5 mM APA (Figure 4 and Figure 6). Moreover, supplementation with Spd restored also the initial morphology and the typical cream color of the colonies and airborne mycelium of the WT strain. Similar, or even more pronounced changes in the WT phenotype were observed when 1 mM concentration of APA was used (Figure S3).

We examined the Put biosynthetic pathway in the HY strain by studying the effects of DFMO and APA. It was found that the HY strain was much less sensitive to either 5 mM DFMO or 5 mM APA, which inhibited the fungal growth by 41% and 44%, respectively, on the 14th day after inoculation (Figure 4 and Figure 5). However, at a lower concentration (1 mM), APA and DFMO were much less effective (Figure S3), which may be attributed to the higher Spd and Spm content in the HY strain (Table 2). 

Next, we studied the effects of the ADC inhibitors on the growth of the *A. chrysogenum* HY strain. Surprisingly, DFMA at 5 mM was only slightly active, inhibiting the fungal growth by only 13% on the 14th day after inoculation (Figure 4 and Figure 5). However, AO-Agm was more potent than DFMA and suppressed the growth of the HY strain by 49% on the 14th day after inoculation (Figure 4 and Figure 5). The good efficacy of AO-Agm might be considered as the first preliminary indication that the HY strain, having an elevated polyamine pool (Table 2), might use both Put biosynthetic pathways (via *L-*Orn and via agmatine) in order to ensure sufficient amounts of the polyamines required for its growth and survival.

## 4. Discussion

ADC belongs to a large family of PLP-dependent decarboxylases, and, like the other PLP-dependent enzymes, it is sensitive to carbonyl reagents (derivatives of hydrazine, hydroxylamine, semicarbazide, etc.), which react with the coenzyme in its active center to inhibit the enzyme. However, simple carbonyl reagents, as a rule, are nonspecific and not very effective inhibitors. All the potent and specific hydroxylamine-containing inhibitors of PLP-dependent ODC [17,18], glutamate decarboxylase [52], gamma-aminobutyric (GABA) transaminase [53], aspartate aminotransferase [54], 1-aminocyclopropane-1-carboxylate synthase [55], γ-cystathionase [56], histidine decarboxylase [57] and pyruvate-dependent AdoMetDC [58,59,60], are structurally similar either to the enzyme substrate or alternatively resemble the product of the enzymatic reaction. The oximes, which are structurally similar to the external aldimines, the first intermediate of PLP-dependent transformations, are formed in the active site of the enzymes. This is the driving force behind the potent and specific inhibition. X-ray studies of the corresponding E-I complexes of aspartate aminotransferase [54], GABA transaminase [53], 1-aminocyclopropane-1-carboxylate synthase [55] and AdoMetDC [60] have confirmed these similarities. Hence, we postulated that AO-Agm, which is an isosteric hydroxylamine analogue of agmatine, would be an effective and specific inhibitor of ADC.

**Figure 7:**
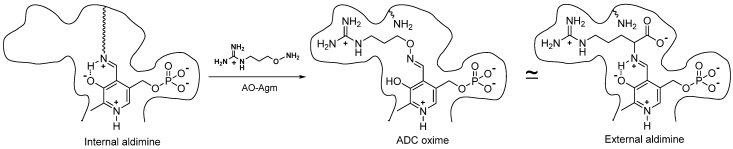
Structures of the internal aldimine, the oxime of ADC with AO-Agm, and the external aldimine—the first intermediate of the decarboxylation of *L-*Arg.

AO-Agm had an *IC*_50_ value of 30 ± 1 nM towards *E. coli* ADC and was a 77 times less potent inhibitor of ODC than of ADC. The guanidino group of AO-Agm anchors the inhibitor in the ADC’s active center, but not as efficiently in the active center of ODC. The properly positioned aminooxy group reacts with the internal aldimine in the enzyme’s active center, giving rise to the PLP oxime, which mimics the external aldimine (Figure 7). This is the reason for the specific and very effective inhibition of ADC. The inhibition of ADC with AO-Agm did not develop with time, as occurs in the case of the inhibition of ODC by APA [17], which is due to the much higher reactivity of Schiff base (internal aldimine) towards *O*-substituted hydroxylamines, if compared with that for PLP itself [46]. On the contrary, the inhibition of pyruvate-dependent *S*-adenosyl-*L*-methionine decarboxylase (AdoMetDC), which possesses a free catalytically crucial carbonyl group in its active center, with a substrate-like *O*-substituted hydroxylamines does develop with time [58,59]. PLP=AO-Agm binds reversibly to ADC i.e., ultrafiltration of ADC~PLP=AO-Agm complex in a PLP-containing buffer fully restored ADC activity, indicating that the inhibition was pseudo-irreversible.

The filamentous fungi have two main biosynthetic pathways which can yield Put. The first and the most ubiquitous pathway consists of the transformation of *L-*Arg into *L-*Orn with the subsequent decarboxylation of the latter compound into Put by ODC. The second, and comparatively rare pathway, involves the transformation of *L-*Arg into agmatine, which is catalyzed by ADC, and the subsequent transformation of agmatine into Put by agmatinase. ODC and ADC are the key and rate-limiting enzymes in these pathways. There are no data in the literature clarifying which of these two pathways of Put biosynthesis is used by *A. chrysogenum*. 

Here, by using an inhibitory analysis we attempted to elucidate which Put biosynthesis pathway is utilized in WT and HY strains of *A. chrysogenum*. The growth of the WT strain was completely inhibited with both of the ODC inhibitors (at 5 mM) while both ADC inhibitors were much less effective (Figure 4 and Figure 6) suggesting that the WT strain of *A. chrysogenum* uses *L-*Orn, but not agmatine as its major precursor of Put. 

The data obtained for the HY strain of *A. chrysogenum* differed from those obtained for the WT strain. First, we found that the HY strain has an elevated polyamine content, as compared with the WT strain (Table 2), i.e., the polyamine biosynthetic machinery was more active. This may be one of the spin-off results of mutagenesis and DNA damage which occurred during the development of the HY strain [37]. It is known that polyamines protect DNA from free-radical damage by reacting direct with the reactive oxygen species [61,62]. Recently it was demonstrated that polyamines can also maintain the genome integrity via homology-directed DNA repair, enhancing the DNA strand exchange activity of RAD51 recombinase. The polyamines stimulate the capture of homologous duplex DNA and promote synaptic complex formation by the RAD51-ssDNA nucleoprotein filament [63]. Therefore, the necessity to have an increased polyamine pool could have been vitally important for *A. chrysogenum* to survive under multi-round mutagenesis, leading to the generation of the HY strain with the elevated polyamine pool. Moreover, the elevated polyamine pool is essential not only for the survival of the fungi during the mutagenesis, but also for the production of secondary metabolites. For example, the addition of exogenous polyamines (1,3-diaminopropane, Spd) increases the production of penicillin G in *Penicillium chrysogenum* Wisconsin 54-1255 strain [64], and the biosynthesis of lovastatin in *Aspergillus terreus* [41,65].

The inhibition of the growth of the HY *A. chrysogenum* with APA and DFMO demonstrated the participation of ODC in the biosynthesis of Put (Figure 4 and Figure 5). The difference in the activities of APA and DFMO towards the HY and WT strains might point to the existence of an additional biosynthetic pathway from *L-*Arg *via* agmatine in the HY strain. Notably, AO-Agm was more potent towards the HY strain, if compared with its activity on the WT strain (Figure 4 and Figure 5). This difference is unlikely to be explained by the nonspecific inhibition of ODC observed in DU145 cells lacking ADC (Table 1) and also in the WT strain (Figure 4 and Figure 5). The good efficacy of AO-Agm may be also considered as the preliminary indication that the HY strain might use also agmatine as a source of Put. Surprisingly, 5 mM DFMA was found to be a poor inhibitor of the growth of the HY strain. (Figure 4 and Figure 5). The low activity of DFMA might indicate that a 5 mM concentration was not enough to inhibit the growth of the HY strain, which has higher concentration of polyamines than the WT strain. Nothing is known about the ADC enzyme present in *A. chrysogenum*, and one cannot exclude the possibility that this enzyme is insensitive to DFMA, resembling the insensitive ODC from *E. coli* [47] and *Entamoeba histolytica* [48] towards DFMO (both DFMA and DFMO are enzyme-activated irreversible inhibitors, having the same mechanism of action). Therefore, the inhibitory analysis provided preliminary evidence that both biosynthetic pathways leading to Put, i.e., via *L-*Orn and via agmatine, might be utilized by HY *A. chrysogenum*. 

## Figures and Tables

**Table 1:** The effects of AO-Agm on the growth and intracellular polyamine pools of DU145 prostate cancer cells. The cells were treated for 3 days with 10–1000 μM AO-Agm in the presence or absence of SC (1 mM). Data are means ± SD, *n* = 3. Statistical significance *, *p* < 0.05; **, *p* < 0.01; *** *p* < 0.001, as compared to the control or SC group. n.d., not detected.

Treatment	Growth	Put	Spd	Spm
	*(Fold)*	*(pmol/mg Protein)*		
Control	1.00 ± 0.01	1395 ± 164	6959 ± 354	3492 ± 175
AO-Agm (10 μM)	0.96 ± 0.02	1515 ± 98	7237 ± 73	3782 ± 55
AO-Agm (100 μM)	0.96 ± 0.04	1447 ± 43	7047 ± 155	3682 ± 152
AO-Agm (1 mM)	0.72 ± 0.04 ***	1074 ± 30 *	3609 ± 124 ***	3795 ± 98
SC	1.00 ± 0.04	1258 ± 157	6483 ± 282	3582 ± 146
SC+AO-Agm (10 μM)	0.94 ± 0.03	922 ± 60 *	6405 ± 61	3903 ± 72
SC+AO-Agm (100 μM)	0.97 ± 0.02	954 ± 115 *	6280 ± 122	3621 ± 87
SC+AO-Agm (1 mM)	0.45 ± 0.01***	n.d. ***	1074 ± 159 ***	2389 ± 105 ***

**Table 2:** The polyamine content in the WT and the HY *A. chrysogenum* strains. The fungi were grown for 24 h as described in Materials and Methods. Data are means ± SD, *n* = 3. Statistical significance, *** *p* < 0.001, as compared with the WT strain, and ^###^ *p* < 001, as compared with the control group of the same strain.

Strain	Addition	Put	Spd	Spm
		*(pmol/mg Dry Weight)*		
WT	Control	<10	670 ± 110	220 ± 30
	5 mM DFMO	<10	140 ± 10	170 ± 20
	0.5 mM Spd	<10	12,200 ± 2180 ^###^	70 ± 10 ^###^
	5 mM DFMO + 0.5 mM Spd	<10	11,530 ± 3690 ^###^	90 ± 30 ^###^
HY	Control	<10	3460 ± 520 ***	990 ± 50 ***
	5 mM DFMO	<10	410 ± 60	424 ± 39 ^###^
	0.5 mM Spd	<10	39,180 ± 4840 ^###^	330 ± 10 ^###^
	5 mM DFMO + 0.5 mM Spd	<10	46,630 ± 6700 ^###^	340 ± 56 ^###^

## Supplementary Materials

The following are available online at https://www.mdpi.com/2218-273X/10/3/406/s1. Figure S1. Measured kinetic parameters for *E. coli* (a) ADC and (b) ODC in the partially purified enzyme preparation. Figure S2. Inhibition (%) of mouse kidney ODC by AO-Agm (reaction conditions: 15 min preincubation of the enzyme with inhibitor in the absence of *L-*Orn; 0.4 mM PLP; reaction was started upon the addition of 0.4 mM *L-*Orn (= 4 × *K_m_*)). Data are means ± SD, *n* = 3. Figure S3. Growth inhibition (%) of the WT and HY *A. chrysogenum* strains with DFMO (1 mM and 5 mM), or APA (1 mM and 5 mM) and the reverse of the inhibition with 0.5 mM Spd at 17 days after inoculation on CD medium.
