# Supplementary material for: Hydroxylamine Analogue of Agmatine: Magic Bullet for Arginine Decarboxylase
Source: Biomolecules. 2020 Mar 6;10(3):406. doi: 10.3390/biom10030406 (PMC7175277; doi:10.3390/biom10030406)
Supplement: Supplementary file 1 [file biomolecules-10-00406-s001.pdf]

__Supplementary Material__

Hydroxylamine Analogue of Agmatine: Magic Bullet for Arginine Decarboxylase

Mervi T. Hyvönen 1*, Tuomo A. Keinänen 1, Gulgina K. Nuraeva 2, Dmitry V. Yanvarev 3, Maxim Khomutov 3, Elena N. Khurs 3, Sergey N. Kochetkov 3, Jouko Vepsäläinen 1, Alexander A. Zhgun 2, Alex R. Khomutov 3*

[1] School of Pharmacy, Biocenter Kuopio, University of Eastern Finland, Kuopio Campus, P.O. Box 1627 Kuopio, FI-70211 Finland;
[2] Research Center of Biotechnology, Russian Academy of Sciences, 119071 Moscow, Russia;
[3] Engelhardt Institute of Molecular Biology, Russian Academy of Sciences, Vavilov Street 32, 119991 Moscow, Russia

Running title: Nanomolar inhibitor of ADC

*To whom correspondence should be addressed:

Mervi T. Hyvönen E-mail: mervi.hyvonen@uef.fi

Alex R. Khomutov. E-mail: alexkhom@list.ru

Contents

Figure 1S. Kinetic parameters for E. coli (A) ADC and (B) ODC S2

Figure 2S. Inhibition of mouse kidney ODC by AO-Agm S2

Figure 3S. Inhibition of the WT and HY A. chrysogenum growth with 1 mM DFMO, or 1 mM APA

.... S3

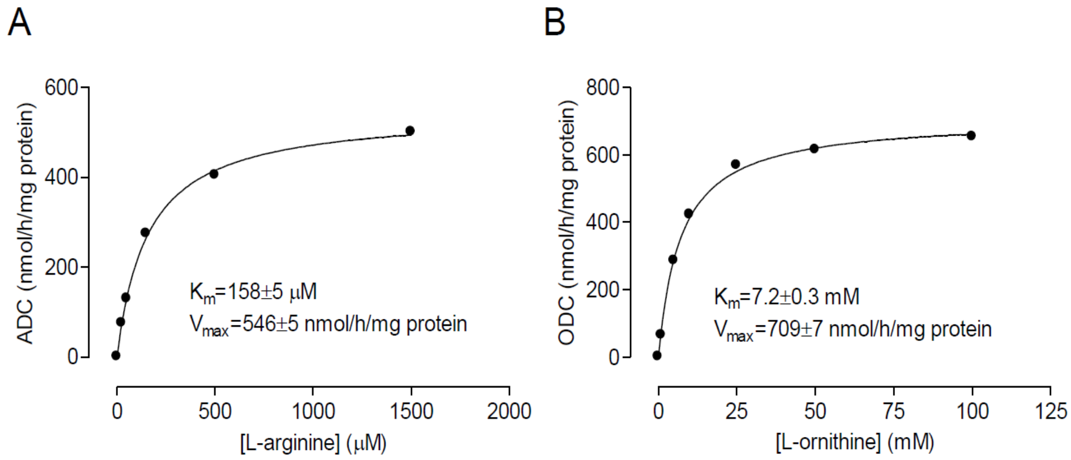

Supplementary Figure S1. Measured kinetic parameters for *E. coli* **(A)** ADC and **(B)** ODC using partially purified enzyme preparation.

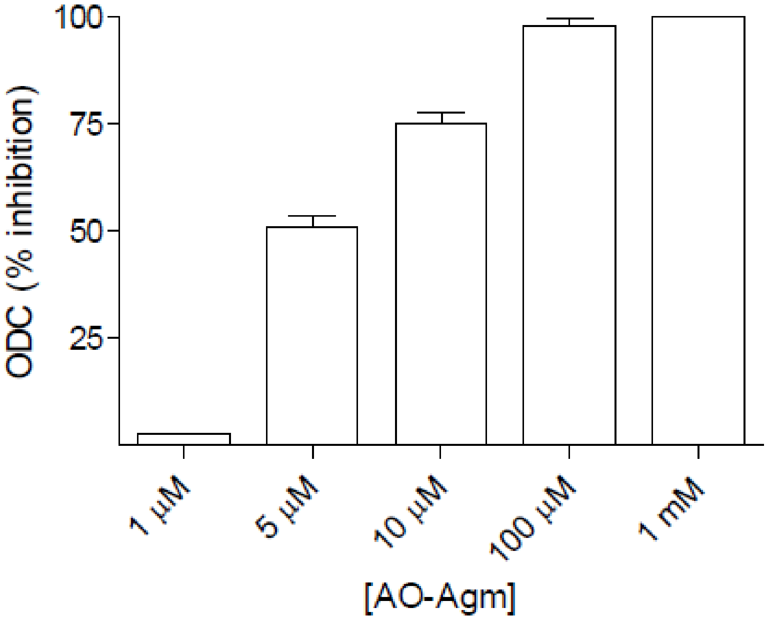

.

Supplementary Figure S2. Inhibition (%) of mouse kidney ODC by AO-Agm (reaction conditions: 15 min preincubation of the enzyme with inhibitor in the absence of *L*-Orn; 0.4 mM PLP; reaction was started upon the addition of *L*-Orn 0.4 mM *L*-Orn (= 4 x *Km*)). Data are means ± SD, *n*=3.

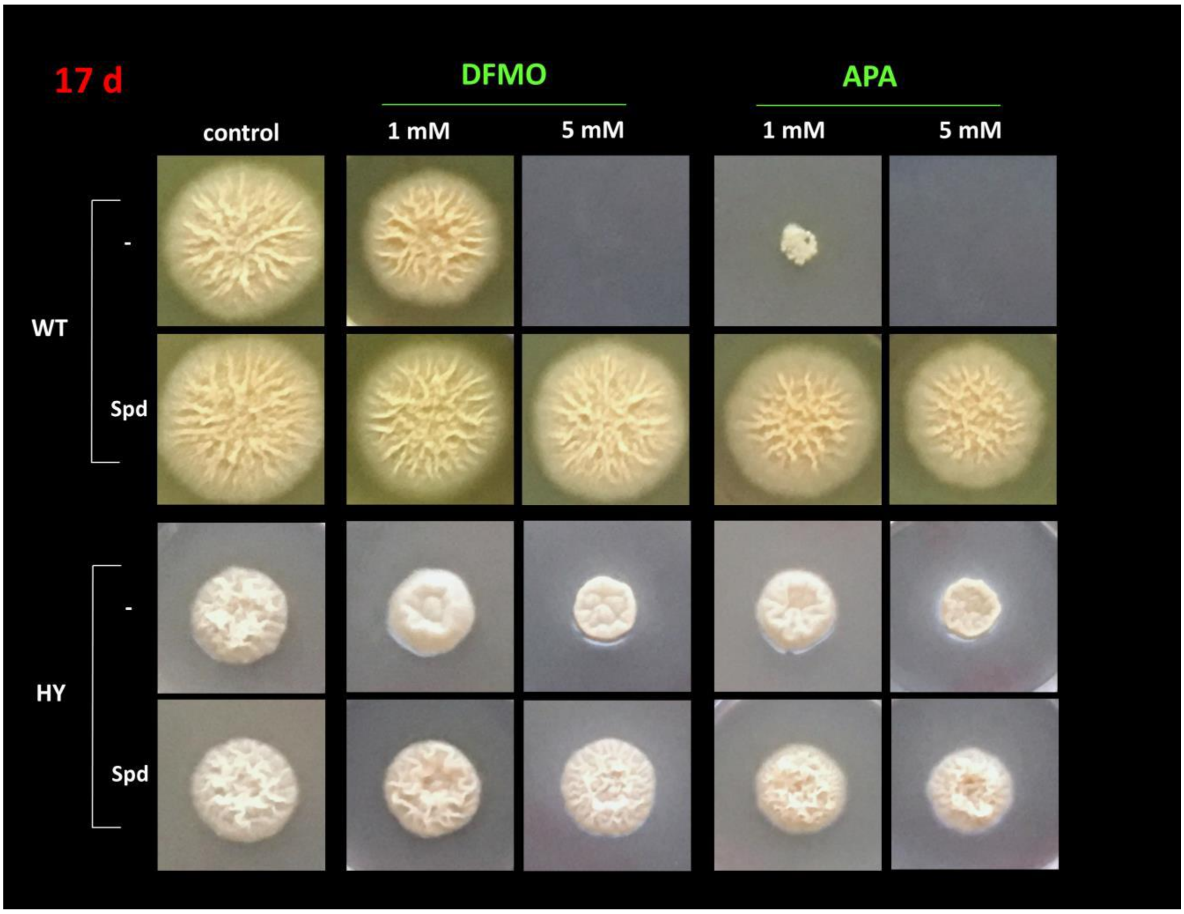

Supplementary Figure S3. Inhibition of the growth of WT and HY *A. chrysogenum* with DFMO (1 mM and 5 mM), or APA (1 mM and 5 mM) and the reversal of the inhibition with 0.5 mM Spd.
